# Postharvest Quality and Storage Stability of Garlic Cultivars From a Highland Semi‐Arid Region

**DOI:** 10.1111/1750-3841.71174

**Published:** 2026-06-20

**Authors:** Juliana Araújo da Silva, Maiara Costa Silva, Marília Alves Brito Pinto, Raquel Cardoso Guimarães, Luís Vicente Lima Teixeira, Sabrina Rocha Silva, Quelmo Silva de Novaes, Gisele Brito Rodrigues

**Affiliations:** ^1^ Southwest Bahia State University Vitória da Conquista Bahia Brazil

**Keywords:** *Allium sativum*, horticulture, physical and chemical quality, postharvest storage

## Abstract

This study aimed to evaluate the impact of storage duration on the physical and chemical properties of garlic bulbs from the Ito and Amarante cultivars, stored in plastic containers under ambient conditions. Temporal analysis was performed using regression models, while differences between cultivars were explored through nonlinear fitting using the LOESS method. Color evaluation was conducted in the CIELAB space, complementing the characterization of physicochemical parameters, including soluble solids, total titratable acidity, pH, pungency, fresh mass loss, and wilting index. In addition, principal component analysis was employed to identify multivariate patterns and correlations among the evaluated variables. The results showed that the Amarante cultivar exhibited greater stability during storage, with a lower wilting index and a gradual increase in soluble solids content. In contrast, the Ito cultivar displayed greater variability, with nonlinear responses and higher susceptibility to wilting, indicating a reduction in quality and commercial value over time. Colorimetric analyses revealed stability in both skin and pulp color in both cultivars throughout storage. Multivariate analysis highlighted the significant influence of genotype on postharvest responses. It is concluded that the Amarante cultivar has greater storage potential and can be stored for up to 90 days, while the Ito cultivar maintains commercial quality for up to 60 days under ambient conditions. These findings contribute to improving postharvest management practices, promoting loss reduction, product valorization, and greater market competitiveness.

## 1 Introduction

Garlic (*Allium sativum*) is a vegetable of significant importance, highly valued for its nutraceutical properties and its role in the global agricultural economy (Lana and Tavares 2010; Feng et al. 2021). Brazil is the world's 16th largest garlic producer (184,844 tons in 2023). The state of Bahia is the leading producer in the Northeast region, with the municipalities of the Chapada Diamantina standing out for an average yield of 10,593 kg ha^−1^ (7881 tons from 744 hectares) and an annual growth rate of 3.6% (Companhia Nacional de Abastecimento [CONAB] 2024; (Instituto Brasileiro de Geografia e Estatística [IBGE] 2024).

Garlic producers in Bahia face intense competition from July to September. The harvest and marketing period for the Bahian crop coincides with the season for garlic from highly technified regions in the Southeast and Central‐West of the country. This concurrent supply surge leads to a decline in market prices, forcing small and medium‐sized local producers to sell their garlic at significantly lower values. As an alternative, some producers have begun storing a portion of their harvest, even for short periods, as a strategy to postpone sales to times of lower market supply.

In Bahia, the production of premium garlic is predominantly based on the Ito cultivar. However, in certain producing regions, such as Cristópolis, there is a higher concentration of semi‐noble (or common) garlic cultivars, particularly Amarante, along with smaller‐scale production of Cateto Roxo. In the “Vale do Alho,” located in the municipality of Novo Horizonte, limited cultivation of the semi‐noble cultivar Catiguá, Gigante Lavínia, and Gigante Roxo is also observed.

Regarding storage, premium garlic bulbs are generally placed in plastic containers after trimming and cleaning, then stacked densely in sheds or open storage structures under ambient conditions. This empirical method is widely adopted by local farmers. In contrast, semi‐noble cultivars are stored in braided strings (ristras) arranged on wooden pallets inside storage facilities. Although these practices are widely adopted due to garlic's inherent capacity to tolerate prolonged storage periods (Oliveira et al. 2004), there remains a scarcity of information regarding the ideal ambient storage conditions. Crucially, the maximum storage duration before bulb quality and market‐valued characteristics are compromised is not well‐defined.

Changes occurring during storage are influenced by factors such as temperature, relative humidity, and packaging type, and may result in losses exceeding 40% under ambient conditions (Madhu et al. 2019; Tripathi et al. 2006). Under appropriate conditions, garlic can be stored for up to 2 months at temperatures between 20°C and 30°C and relative humidity below 75%, although losses in firmness and changes in color may still occur (Saif et al. 2020; Madhu et al. 2019).

The main factors associated with deterioration during storage involve physical and chemical changes, typically assessed through qualitative analyses. Among these, colorimetric evaluations stand out, as well as the determination of pH, SS, TTA, Pg, FML, and WI (Chitarra and Chitarra 2005; Lucena et al. 2016; Pardo et al. 2007). The WI is a serious postharvest defect in garlic, characterized by the loss of clove firmness during storage. According to Brazilian Ministry of Agriculture Ordinance No. 435/2022, its incidence is limited to 2%, 3%, and 4% of bulbs for the Extra, Class I, and Class II categories, respectively, thereby directly influencing the product commercial classification.

Given the increasing market value of garlic and the need to extend its shelf life, this study aimed to evaluate the effect of ambient storage in a high‐altitude region of Bahia state on the physical, chemical and colorimetric characteristics of the (I) and (A) cultivars.

## 2 Materials and Methods

### 2.1 Study Location and Plant Material

The experiment was conducted at the State University of Southwest Bahia (Universidade Estadual do Sudoeste da Bahia—UESB), Vitória da Conquista campus (14°53′ S, 40°48′ W), between October 2024 and March 2025. Bulbs of the (A) (semi‐noble) and (I) (noble) garlic cultivars were obtained from a certified producer in the municipality of Cristópolis and from an experimental field at UESB. The bulbs were graded according to the standards established by MAPA (Ministry of Agriculture, Livestock and Supply) Normative Instruction N° 435 of 2022 (Brasil 2022), falling into Categories 5 (46–50 mm diameter) and 6 (51–55 mm diameter). The bulbs were stored in polypropylene boxes under ambient conditions, in the dark, with airflow provided by a timer‐controlled air cooler. Temperature and relative humidity were continuously monitored using a data logger (Hobo UX100‐011, Bourne, USA). Throughout the storage period, partial environmental control strategies were applied, including forced ventilation and the placement of water‐filled containers, to reduce microclimatic fluctuations.

### 2.2 Experimental Design

A completely randomized design (CRD) was employed in a 2 × 5 factorial arrangement with twelve replications. The factors consisted of two cultivars (I) and (A) and five storage periods (0, 30, 60, 90, and 120 days). Each experimental unit consisted of five bulbs.

### 2.3 Sample Preparation

At each storage interval, bulbs were selected based on criteria of physical integrity, including the absence of visible microbial contamination or mechanical damage, typical coloration, and firmness to the touch. The cloves were manually separated from the bulb, peeled, and processed in a grinder (Britânia/BMP900P, Joinville, Brazil) for 30 s. The resulting material was then homogenized in a mixer (Mondial/M‐15‐W, Barueri, Brazil) for 30 s at medium speed. The obtained flesh was packaged in 140 mL polyethylene terephthalate (PET) containers and maintained in a climate‐controlled room at approximately 25°C for subsequent analyses.

### 2.4 Chemical Analysis

- *Soluble Solids (SS)*: Measured using a digital refractometer (Soonda, China) calibrated with distilled water. Analyses were performed in triplicate.
- *pH*: Determined by direct potentiometric method using a digital pH meter (Adamo/mPA‐210, Piracicaba, Brazil) previously calibrated with standard pH 4.0 and 7.0 solutions, according to the Association of Official Analytical Chemists (2016).
- *Total Titratable Acidity (TTA)*: Determined by titrating 1 g of flesh diluted in 50 mL of distilled water, using 0.1 N NaOH and phenolphthalein as an indicator (IAL 1985). Results were expressed in mEq mL^−1^.
- *Pungency (Pg)*: Quantified by determining pyruvic acid content via spectrophotometry at 420 nm, using 2,4‐dinitrophenylhydrazine (DNPH), according to Anthon and Barrett (2003) with adaptations from Schwimmer and Watson (1961). Results were expressed in µmol mL^−1^ of garlic extract. The determination of Pg based on pyruvic acid content constitutes the most established and widely used method for evaluating garlic Pg, as reported by El‐Mesery et al. (2025).

### 2.5 Physical Analysis

- *Fresh Mass Loss (FML)*: Calculated as the percentage difference between the initial and final fresh mass.
- *Wilting Index (WI)*: Calculated as the proportion of withered cloves relative to the total number of cloves per bulb (%).

### 2.6 Colorimetric Determination

Color measurements (CIELAB system) were obtained using a portable colorimeter (Linshang LS173 D/8, China) calibrated with a white tile standard. For each treatment, three readings were taken on the central region of both the clove's outer skin and the flesh, totaling 36 measurements per cultivar and storage period. Based on the *L, a*, and b* values, the hue angle (h°) and chroma were calculated as described by McGuire (1992), using the following adapted equations:

$$\mathrm{Hue}=\left(\left(ArcTAN\left(b^{*}/a^{*}\right)2\pi \right)\times 360\right)$$

$$Chroma=\left({\left(a^{2}+b^{2}\right)}^{1/2}\right)$$

### 2.7 Statistical Analysis

Statistical analyses were conducted according to the characteristics of the data. To evaluate the behavior of the cultivars over the storage period, regression models were fitted to describe the temporal trends of the variables. Model selection was based on the observed behavior of the samples, accounting for potential nonlinear responses commonly found in biological data influenced by factors such as temperature and humidity. Model adequacy was assessed using the coefficient of determination (*R* ^2^) and the statistical significance of the coefficients, enabling the selection of models that best captured the dynamics of the variables.

LOESS (Locally Estimated Scatterplot Smoothing) regression was applied to assess cultivar behavior, as it is a nonparametric method that fits smooth curves without assuming a linear relationship between variables. This approach allows the identification of general trends and nonlinear patterns, providing a more flexible representation of the data.

Principal component analysis (PCA) was used to reduce data dimensionality and to explore patterns of association among variables and samples. The biplot enabled the joint visualization of these relationships, facilitating the interpretation of correlations and variable contributions. Ninety‐five percent confidence ellipses were included to represent group dispersion and to aid in assessing treatment separation, providing a more robust visual interpretation of the data. The data were standardized, and the PCA was conducted using the maximum likelihood method.

For the color parameters (L*, a*, b*, Chroma, h°), a nonparametric approach was adopted after the assumptions of normality and homogeneity of variances were not met, as verified by Shapiro–Wilk and Levene's tests, respectively. This approach ensured greater robustness and reliability in the comparisons.

All analyses were performed using R software (version 4.5.1; R Core Team, 2025).

## 3 Results

### 3.1 Environmental Monitoring

**FIGURE 1:**
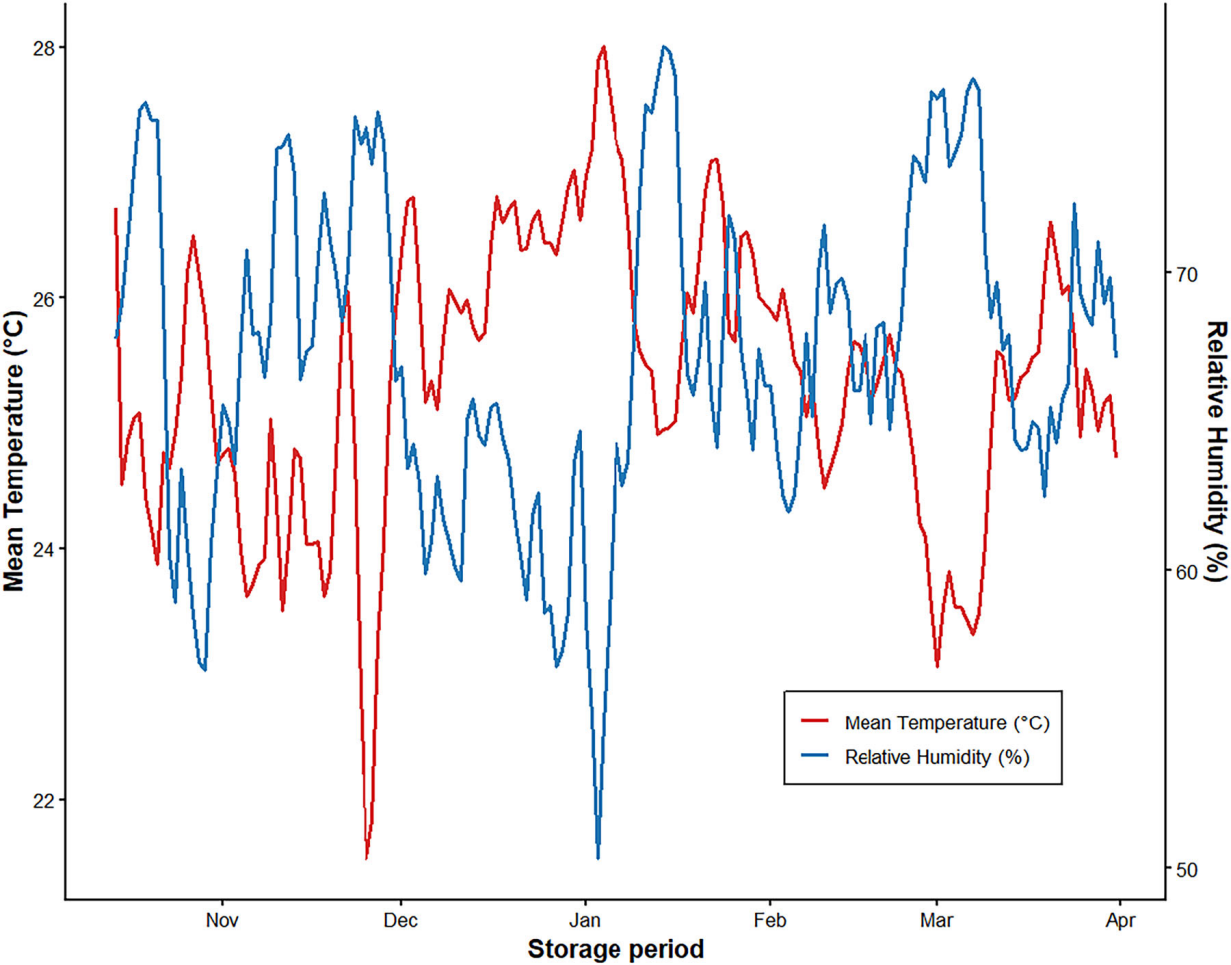
Relative humidity (RH%) and mean temperature (°C) recorded from October, 2024 to March, 2025.

As shown in Figure 1, the monthly average relative humidity during the experimental period ranged from 62.16% to 70.32%. In October 2024, the average humidity was 66.20%, reaching its maximum value in November (70.32%). A pronounced reduction was observed in December and early January, with values of 62.16% and 52%, respectively, followed by a gradual increase: from the second half of January (67.11%) to February (68.02%), and March 2025 (69.36%). Regarding the average temperature, values fluctuated between 24.18°C and 26.30°C. The lowest temperature was recorded in November 2024 (24.18°C), while the highest values occurred in December (26.26°C) and January (26.30°C). These fluctuations in storage environment humidity and temperature throughout the experiment are relevant, as they can affect key physiological processes in the bulbs, such as respiration, water loss, and overall quality maintenance during the storage period (Sohany et al. 2016).

### 3.2 Regression Analysis for the Storage Period

Regression analysis was employed to evaluate changes in the dependent variables over the storage period, identifying distinct performance patterns between the cultivars (Table 1).

**TABLE 1:** Regression models and statistical parameters of the physicochemical variables evaluated in the Amarante (A) and Ito (I) cultivars.

Cultivar	Variable	Model fit	*R* ^2^	*F*	*p*‐value
**A**	**SS**	Linear	0.035	2.151	0.148
	**TTA**	Quadratic	0.560	36.289	< 0.001
	**pH**	Cubic	0.883	31.045	< 0.001
	**Pg**	Linear	0.624	61.175	< 0.001
	**FML**	Linear	0.678	117.829	< 0.001
	**WI**	Linear	0.063	1.953	0.168
**I**	**SS**	Cubic	0.524	12.2209	< 0.0001
	**TTA**	Cubic	0.478	15.226	< 0.0001
	**pH**	Linear	0.935	427.910	< 0.001
	**Pg**	Quadratic	0.498	28.254	< 0.001
	**FML**	Linear	0.466	19.499	< 0.001
	**WI**	Linear	0.128	8.581	0.050

*Note*: Significance level adopted: *p* < 0.05.

Abbreviations: *F*: *F*‐test value; *p*: probability associated with the *F*‐test; *R* ^2^: coefficient of determination.

Overall, the (A) cultivar exhibited statistically significant trends (*α* = 0.05) for most variables, with a predominance of linear models. This indicates progressive and consistent changes over time, suggesting that the parameters for this cultivar change in a predictable manner throughout storage without major fluctuations.

In contrast, the (I) cultivar displayed a greater diversity of fitted models, including quadratic and cubic trends. The cubic regressions for variables such as SS and TTA reveal a more complex pattern of variation, characterized by fluctuations that may be linked to dynamic physiological processes. These processes are potentially influenced by cultivar‐specific traits or their interaction with the storage conditions.

The pH variable, which showed a strong linear fit for (I), suggests a clear and continuous directional change over time. This trend may indicate either a stable degradation process or the accumulation of specific compounds influencing acidity. Conversely, the WI was not significantly affected by storage time in either cultivar.

**FIGURE 2:**
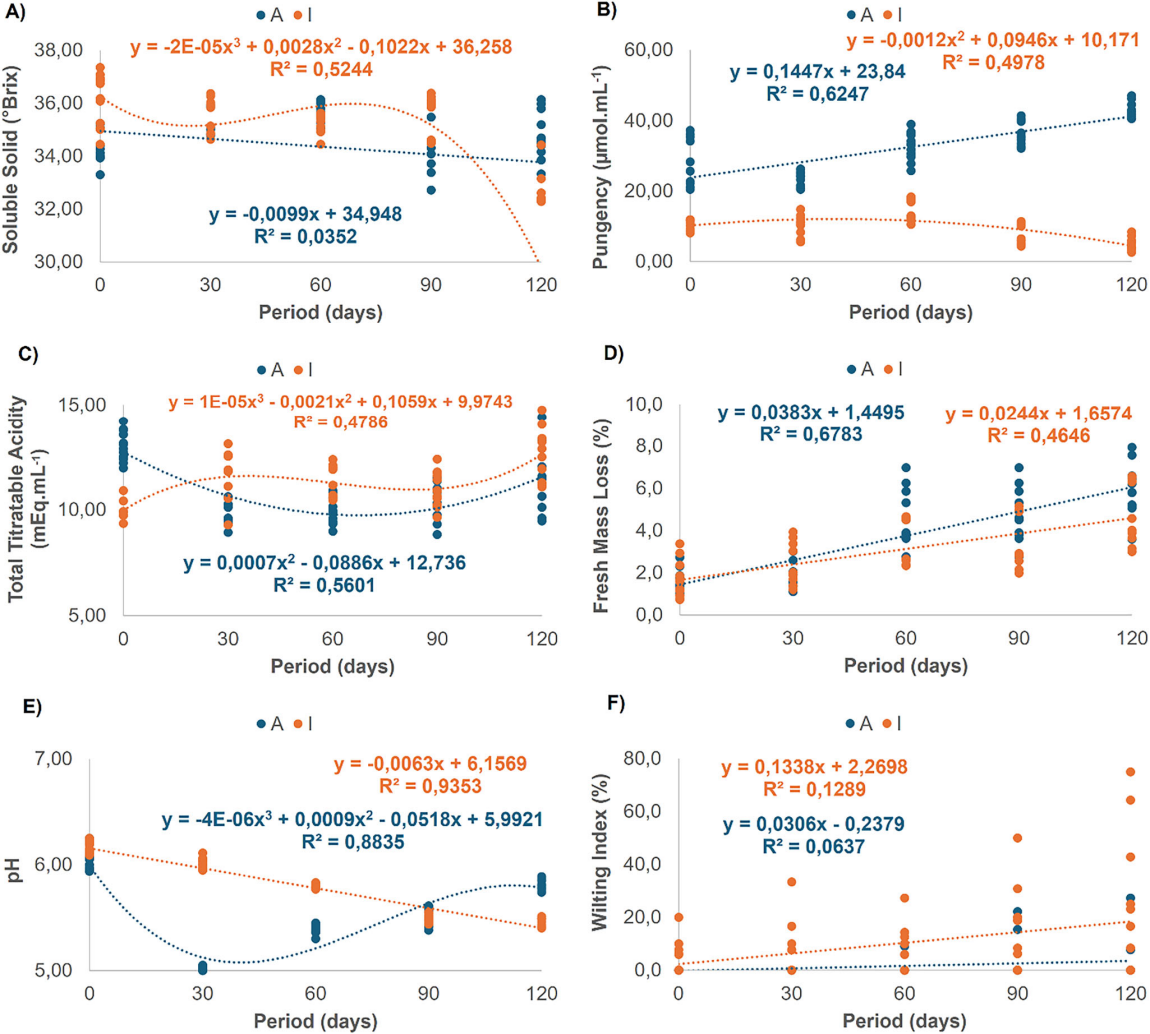
Soluble solids (A), pungency (B), titratable acidity (C), mass loss (D), pH (E), and wilting index (F) of garlic cultivars. Amarante (A) and Ito (I) stored for 120 days under ambient conditions.

In Figure 2A, the SS content of cultivar (A) exhibited a linear trend with no statistically significant differences, indicating no variation over the 120 days of storage. In contrast, the (I) cultivar showed a significant fit to a cubic model. This indicates an initial increase in SS content until approximately 90 days, followed by a subsequent decrease, suggesting potential degradation; however, as the model explains only 52.4% of the observed variation, other factors—such as environmental and crop management conditions—are indicated as having a direct influence on garlic bulb degradation.

The Pg variable demonstrated distinct behavior between the evaluated cultivars. The (A) cultivar maintained higher Pg values than the (I) cultivar, indicating better preservation of quality and intrinsic bulb characteristics during storage. In contrast, the (I) cultivar exhibited a trend of compound degradation over time (Figure 2B). For the (A) cultivar, a constant increase in Pg was observed throughout the storage period, starting from approximately 24 µmol mL^−1^ and reaching values near 45 µmol mL^−1^ at 120 days. Conversely, the (I) cultivar showed a quadratic fit, with an initial increase in Pg up to 60 days, followed by a reduction after 90 days of storage, reaching a minimum value of 4.95 µmol mL^−1^ around 120 days.

As shown in Figure 2C, the variation in TTA for the (I) cultivar was best described by a cubic model, showing an increase in acidity up to 120 days. In contrast, the (A) cultivar was better fitted by a quadratic model, characterized by an initial decrease until approximately 60 days, followed by a gradual increase until the end of the storage period. For the pH variable (Figure 2E), the (I) cultivar exhibited a linear behavior, with a continuous downward trend in pH over the storage period, indicating progressive acidification—an inverse pattern to its TTA. Conversely, the (A) cultivar displayed a quadratic fit, a result consistent with its TTA profile, in which the pH decreased until 30 days and then returned to near‐neutral levels at 90 and 120 days.

The FML increased linearly throughout the storage period for both cultivars (Figure 2D). For the (A) cultivar, the linear model provided the best fit (*R* ^2^ = 0.6783), with an average daily increase of 0.0383%, rising from 1.4% mass loss at day 0%–7% at 120 days. Similarly, for the (I) cultivar, the linear model best explained the mass loss (*R* ^2^ = 0.4668), albeit with a slightly lower daily loss rate of 0.0288%. Overall, the (A) cultivar exhibited higher mass loss values throughout storage, suggesting lower water retention and higher bulb transpiration rates. These results demonstrate that, regardless of the cultivar, storage promotes progressive losses in the fresh weight of garlic bulbs, associated with the reduction of moisture content and ongoing tissue respiration.

When evaluating the effect of storage time, a gradual increase in the WI was observed for both cultivars, despite high data variability and poor model fits (*R* ^2^ < 0.13). The linear model was considered the most appropriate for describing this trend, indicating a general increase in WI over the storage period (Figure 2F). The (A) cultivar showed no withered cloves until 30 days, reaching the maximum limit permitted by ministerial regulations (4%) at 60 days and exceeding this threshold only after 90 days. In contrast, the (I) cultivar exhibited values above the limit from the outset, with a marked and continuous increase throughout storage, far exceeding the recommended values by 120 days. Overall, the temporal progression of the WI indicates that prolonged storage promotes clove wilting, with more stable behavior observed only during the initial storage periods.

### 3.3 Cultivar Comparison Using LOESS Regression

**FIGURE 3:**
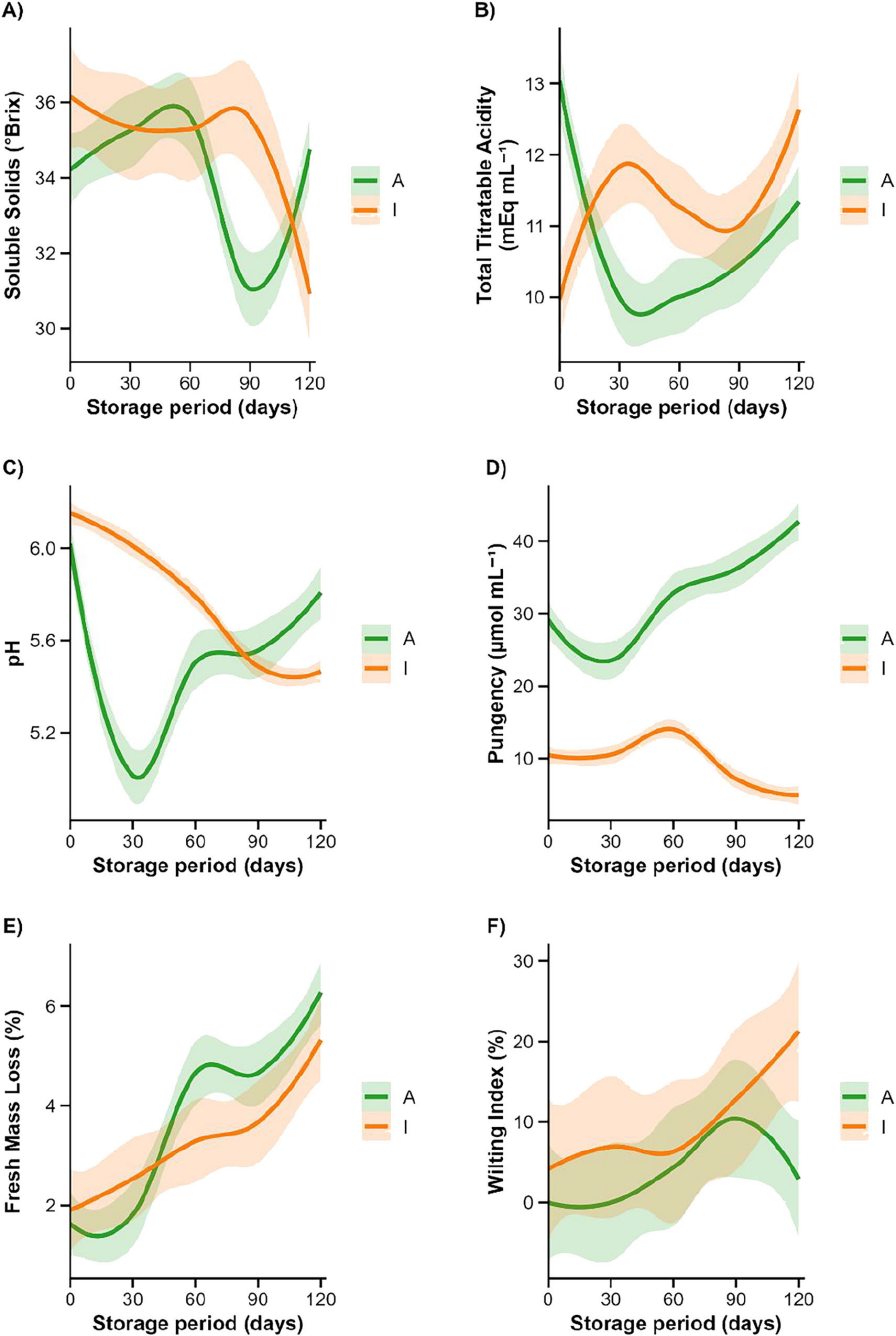
LOESS regression of the variables: (A) soluble solids (°Brix, SS); (B) total titratable acidity (mEq mL^−1^, TTA); (C) pH; (D) pungency (µmol mL^−1^, Pg); (E) fresh mass loss (%, FML); and (F) wilting index (%, WI) in garlic cultivars Amarante (A) and Ito (I) during the storage period (0, 30, 60, 90, and 120 days).

LOESS smoothing analysis revealed differences in the behavior of the cultivars throughout storage. Overall, cultivar (I) exhibited higher average SS levels for most of the evaluated period, while cultivar (AB) maintained slightly lower values, with greater variation across observations. Despite some localized variations, cultivar (I) displayed a more stable trend, whereas (AB) showed more pronounced fluctuations, suggesting inherent differences between the cultivars in maintaining SS levels under storage conditions (Figure 3A).

TTA exhibited distinct behavior between the cultivars (Figure 3B). Cultivar (AB) started with high acidity values, followed by a sharp decrease until approximately 30 days, after which it maintained a gradual increase until the end of the evaluations. In contrast, cultivar (I) showed the opposite pattern, with an increase in acidity values throughout storage. These differences indicate that the cultivar factor strongly influenced the variation in acidity. The pH variable varied inversely with TTA (Figure 3C) for cultivar (I), which maintained an acidification trend with decreasing pH values during the experiment. In contrast, cultivar (AB) showed a sharp drop in the first 30 days, followed by a gradual increase until the end of storage. The opposite behavior between TTA and pH confirms the inverse relationship between these two variables, reflecting the acid–base balance of the bulbs during storage for cultivar (I). However, this pattern was not observed for cultivar (A).

Garlic bulb Pg was strongly affected by the cultivar during the storage period (Figure 3D). Cultivar (A) consistently exhibited substantially higher Pg values than cultivar (I) throughout the entire storage period. In cultivar (AB), Pg showed a slight initial reduction until approximately 30 days, followed by a continuous increase until the end of the 120‐day period. In contrast, cultivar (I) exhibited stable Pg levels during the initial 30 days, followed by a slight increase at 60 days and a subsequent decline. This cultivar accumulated fewer organosulfur compounds, characterizing it as a cultivar with lower Pg intensity. These results demonstrate a clear genotypic effect on Pg. However, the variations observed between the cultivars may have been influenced by environmental factors and prestorage field management practices, which affect the synthesis and accumulation of the sulfur compounds responsible for Pg intensity.

FML increased progressively throughout the storage period in both cultivars, indicating the ongoing processes of bulb transpiration and respiration (Figure 3E). Cultivar (AB) exhibited slightly higher values than those observed in cultivar (I), particularly after 60 days of storage. This pattern suggests that cultivar (AB) is more susceptible to dehydration than cultivar (I). The increasing trend observed in both cultivars is characteristic of produce stored under prolonged conditions, reflecting a gradual reduction in bulb moisture and fresh matter.

The WI increased progressively during the storage period in both cultivars (Figure 3F), with a more pronounced progression in cultivar (I), which reached values close to 25% at 120 days, while cultivar (AB) maintained levels below 15%. This result indicates a greater susceptibility of cultivar (I) to loss of bulb firmness over time. According to Brazilian Normative Instruction MAPA No. 435 of May 18, 2022, the presence of shriveled cloves is considered a serious defect, with tolerances limited according to the product's classification category. Thus, the values observed after 90 days of storage, especially for cultivar (I), exceed the acceptable level for commercial standards, indicating that prolonged storage periods compromise the commercial quality of the bulbs.

### 3.4 Principal Component Analysis

**FIGURE 4:**
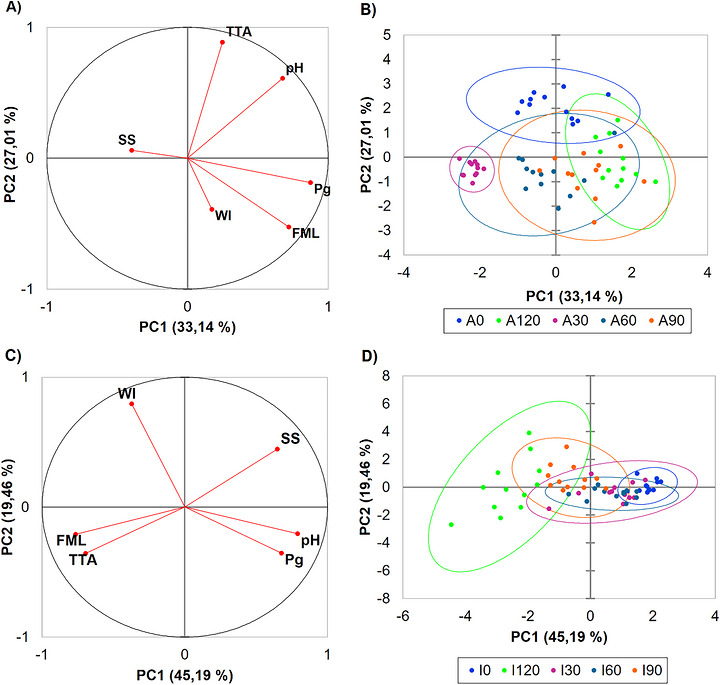
Principal component analysis (PCA) of the physicochemical attributes of garlic cloves from the Amarante (A) and Ito (I) cultivars over five storage periods (0, 30, 60, 90, and 120 days). Panels (A) and (B) show, respectively, the variable biplot and the score distribution with 95% confidence ellipses for the Amarante cultivar. Panels (C) and (D) present the variable biplot and the score distribution with 95% confidence ellipses for the Ito cultivar. The vectors represent the contribution of the analyzed variables: pH, soluble solids (SS), mass loss (FML), titratable acidity (TTA), wilting index (WI), and pungency (Pg), and the values in parentheses on the axes indicate the percentage of variance explained by the principal components.

For the Amarante (A) cultivar (Figure 4A), PCA explained 60.28% of the cumulative variance in the first two components (PC1 = 33.14%, PC2 = 27.01%), whereas for the Ito (I) cultivar (Figure 4C), it accounted for 64.25% (PC1 = 45.19%, PC2 = 19.46%).

For cultivar (A), PCA showed that PC1 was primarily influenced by pH, Pg, and FML, indicating that samples with higher FML, Pg, and pH values tend to exhibit a slight reduction in SS. This pattern reveals an inverse relationship among these attributes, suggesting that increased mass loss is associated with enhanced Pg and a possible decline in sample sweetness. PC2 was strongly and positively influenced by TTA and pH, and negatively by FML, indicating that samples with higher TTA and pH tend to exhibit moderate FML (Figure 4A, Table 2).

**TABLE 2:** Correlation among variables in the first two principal components (PC1, PC2) for the Amarante (A) and Ito (I) cultivars.

	PC1		PC2	
	(A)	(I)	(A)	(I)
SS	−0.397	0.649	0.060	0.446
TTA	0.249	−0.695	0.885	−0.356
pH	0.675	0.790	0.609	−0.205
Pg	0.875	0.677	−0.186	−0.354
FML	0.719	−0.765	−0.525	−0.209
WI	0.173	−0.373	−0.390	0.794

*Note*: Values close to −1 or 1 indicate a stronger contribution of the variables to the respective component.

Abbreviations: FML, fresh mass loss; Pg, pungency; pH; SS, soluble solids; TTA, total titratable acidity; WI, wilting index.

For cultivar (I), PC1 showed positive correlations with Pg, pH, and SS, and negative correlations with TTA and FML. PC2 displayed a moderate association with SS and a strong positive influence of WI, while the remaining variables showed weak associations, indicating that this component was predominantly explained by variation in the WI. This pattern indicates that samples with higher WI values display a distinct profile relative to the other quality attributes, suggesting a progressive deterioration of physicochemical properties as WI increases (Figure 4C, Table 2).

For cultivar (A), the A0 period was predominantly located in the upper region of the plot, indicating a distinct initial profile compared with the other storage times. The A30 period was displaced relative to the others, with partial overlap with A60, suggesting early changes in sample characteristics after 30 days of storage. Periods A60, A90, and A120 showed substantial overlap, indicating similar physicochemical behavior across these intervals. However, A120 was shifted further toward the positive region of PC1, in contrast to A30, suggesting a gradual modification of sample characteristics as storage progressed. Overall, the main changes occurred within the first 30 days, with reduced discrimination among subsequent periods (Figure 4B).

For cultivar (I), A0, A30, and A60 tended to cluster in the positive region of PC1, indicating a stronger association of these storage periods with Pg, pH, and SS. In contrast, A90 and A120 were predominantly located in the negative region of PC1, closer to the vectors for WI, FML, and TTA. This pattern suggests a progressive shift in the physicochemical profile of the samples during storage, whereby after 90 days, the garlic becomes more strongly associated with attributes indicative of postharvest quality deterioration.

### 3.5 Colorimetric Analysis

The results of the Shapiro–Wilk test indicated that none of the evaluated variables followed a normal distribution (*p* < 0.05). According to Levene's test, homogeneity of variances was observed for chroma and hue of the skin (*p* > 0.05), whereas chroma and hue of the flesh exhibited heterogeneity of variances (*p* < 0.05), as shown in Table 3.

**TABLE 3:** Normality (Shapiro–Wilk) and homogeneity of variances (Levene) tests for the colorimetric variables (Hue and Chroma) of garlic skin and flesh.

	Shapiro–Wilk		Levene	
	*W*	*p*‐value	*F*	*p*‐value
Chroma skin	0.963	0,002	2.340	0.129
Hue skin	0.732	< 0.0001	0.958	0.330
Chroma flesh	0.961	0.001	6.734	0.011
Hue flesh	0.556	< 0.0001	5.514	0.021

*Note: W* and *p*‐values refer to the Shapiro–Wilk test for assessing data normality. *F* and *p*‐values correspond to Levene's test for homogeneity of variances. *p*‐values < 0.05 indicate violation of the assumptions of normality and/or homoscedasticity.

The median values, along with letter groupings indicating statistically significant differences according to the Mann–Whitney test (*p* < 0.05), are presented in Table 4 for the color variables of garlic clove skin and flesh over the storage period (0, 30, 60, 90, and 120 days).

**TABLE 4:** Medians of Hue (H) and Chroma (C) variables, with letter groupings indicating statistical differences according to the Mann–Whitney test (*p* < 0.05) for skin data of the Amarante (A) and Ito (I) cultivars during the storage period (0, 30, 60, 90, and 120 days).

	Skin		Flesh	
	H	C	H	C
A0	63.38 ± 19.1ab	10.87 ± 2.37ab	98.2 ± 9.0abcd	12.9 ± 3.6abc
A30	65.58 ± 5.32a	12.16 ± 2.45ab	94.3 ± 2.62a	19.2 ± 3.40a
A60	50.97 ± 7.42bc	9.02 ± 0.93a	98.7 ± 0.80b	12.8 ± 1.38bc
A90	67.40 ± 5.85a	11.73 ± 1.27b	98.3 ± 5.6bcd	12.0 ± 1.39b
A120	62.10 ± 10.57ab	10.00 ± 1.58ab	97.2 ± 0.97ac	15.3 ± 1.82ac
I0	14.22 ± 33.6cd	18.97 ± 3.58c	98.85 ± 0.89b	14.4 ± 0.85c
I30	24.93 ± 16.85cd	17.41 ± 1.39c	97.4 ± 1.4abcd	13.2 ± 2.17bc
I60	18.15 ± 6.58d	19.58 ± 2.93c	98.2 ± 0.50bd	14.6 ± 0.67c
I90	21.45 ± 9.31d	19.29 ± 2.10c	99.0 ± 1.41bcd	13.5 ± 1.32bc
I120	17.70 ± 13.78d	19.32 ± 2.89c	97.0 ± 0.8cd	13.8 ± 1.94bc

*Note*: Medians followed by the same letter in the column do not differ significantly from each other according to the multiple comparisons test (*p* < 0.05).

#### 3.5.1 Skin

##### 3.5.1.1 Chroma (Saturation)

The (A) cultivar exhibited chroma values ranging from 9.02 to 12.16, showing minor variations and some statistical differences throughout storage, as indicated by the letter groupings “a.” “b.” and “ab” (Table 4). This suggests that the intensity of the skin color underwent a few changes. In contrast, the (I) cultivar displayed a consistently higher chroma, ranging from 17.41 to 19.58, with no significant statistical differences among the storage periods (Group “c”). This indicates that this cultivar had a more intense skin color, which remained stable throughout storage without significant darkening. In summary, (I) exhibited greater color saturation than (A) across all evaluated periods.

##### 3.5.1.2 Hue (Tonalidade)

For the (A) cultivar, hue values ranged from 50.97 to 67.40. The A0 treatment did not differ statistically from the A30, A90, and A120 periods, sharing the same statistical grouping “a”. However, a significant difference was observed for the A60 treatment, which, although distinct from the other periods, did not differ statistically from A120 (sharing group “b”) (Table 3). In contrast, the (I) cultivar recorded significantly lower hue values, ranging from 14.22 to 24.93 across all treatments. The I0 and I30 treatments were grouped into category “cd,” along with the A60 treatment. From the 60‐day period onward (I60), the (I) cultivar treatments (I60, I90, and I120) remained in Group “d” until the end of storage (Table 4).

**FIGURE 5:**
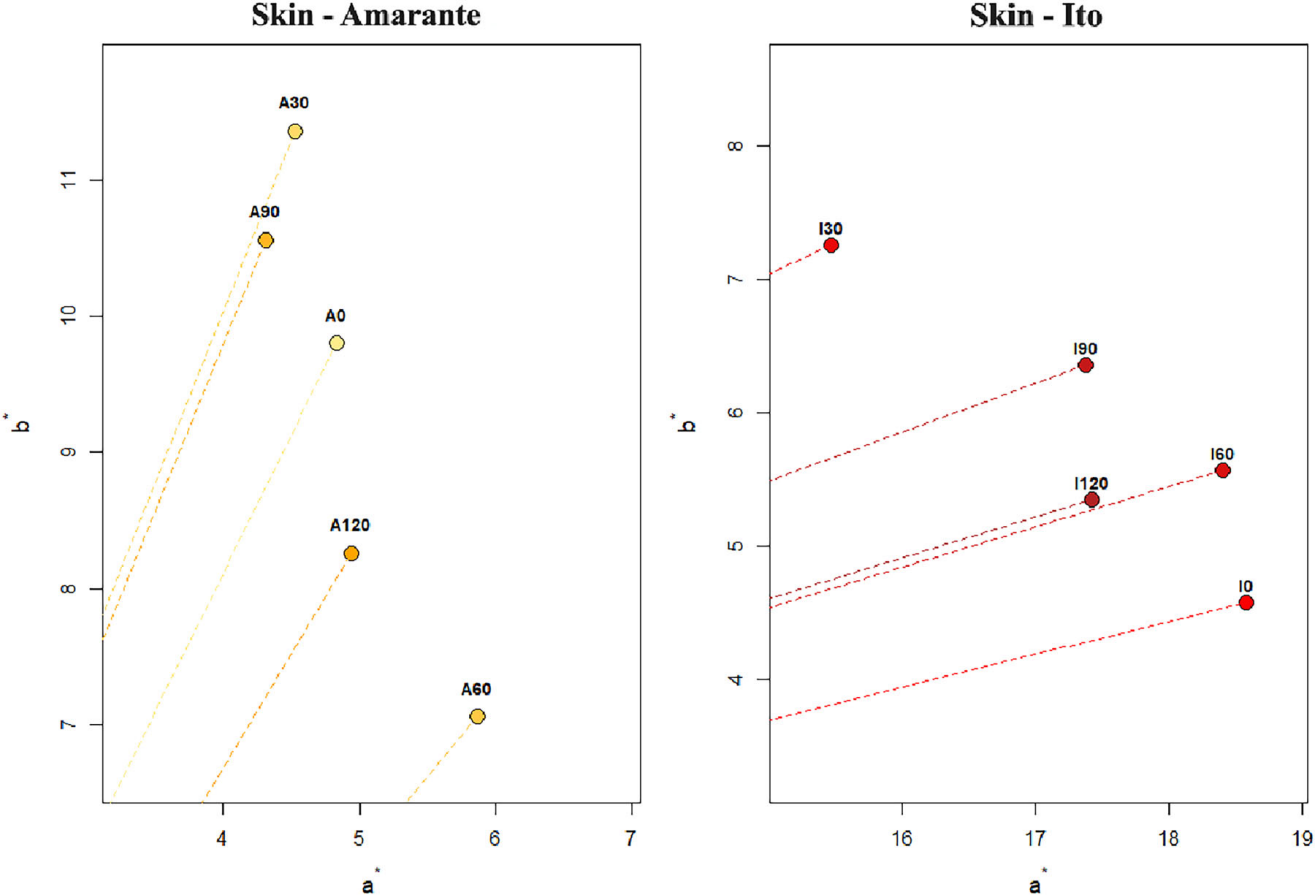
Median coordinates converted to RGB for the skin of the Amarante (A) and Ito (I) cultivars over the time interval (0, 30, 60, 90, and 120 days) on the a* and b* axes of the CIELAB color space.

The (A) cultivar maintained a significantly higher hue, with a predominant tendency towards the b* axis (positive values), characterizing a yellowish coloration throughout storage. In contrast, the (I) cultivar exhibited consistently lower hue values, with a tendency towards the a* axis (positive values), indicating a predominant intense red coloration in all periods, as can be observed in Figure 5.

#### 3.5.2 Flesh

##### 3.5.2.1 Chroma (Saturation)

As shown in Table 4, the chroma values of the flesh ranged from 12.01 to 19.14, with a significant interaction between cultivars and time. The A30 treatment exhibited the highest chroma value (19.14, group “a”), indicating a more saturated color at this stage. However, it did not differ statistically from treatments A0 (“abc”) and A120 (“ac”), which share the predominant grouping “a”. The A90 treatment (group “b”) showed the lowest chroma value and did not differ statistically from groups A60 (“bc”), I30 (“bc”), I90 (“bc”), and I120 (“bc”). These groups show overlapping letters but a predominance of group “b”. The positions of the CIELAB coordinates and the flesh chroma can be observed in the corresponding figure (Figure 5).

##### 3.5.2.2 Hue (Tonality)

The flesh Hue values remained high, ranging from 94.26 to 98.97, indicating yellowish/greenish tones in the flesh of both cultivars. The (A)  cultivar exhibited slightly lower values (94.26–98.73) compared to (I) (97.20–98.97), with the exception of the A30 treatment (94.26), which was significantly lower, indicating a tone closer to a darker yellow (Table 4).

For the (I) cultivar, the values were more stable, with no pronounced differences over time, and a predominance of group “b” for the treatments I0 (“b”), I30 (“abcd”), I60 (“bd”), and I90 (“bcd”). The I120 treatment recorded the lowest Hue value for the (I) cultivar (97.20, group “cd”), differing statistically from the I0 treatment, which indicates a darkening of the tone at this stage. Conversely, it showed no significant difference from treatments A0 (“abcd”), A90 (“bcd”), and A120 (“ac”) (Table 4).

**FIGURE 6:**
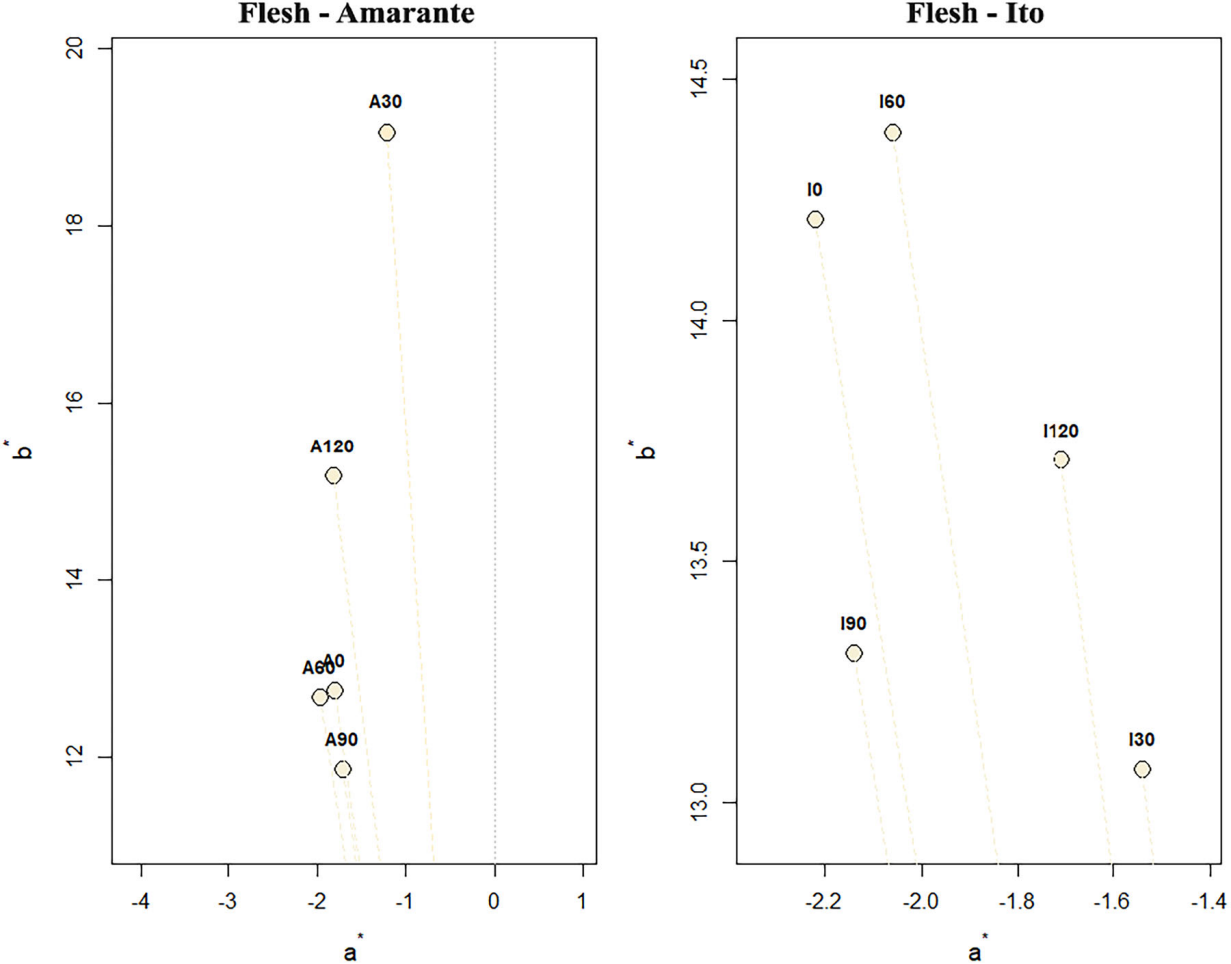
Median coordinates converted to RGB for the pulp of the Amarante (A) and Ito (I) cultivars over the time interval (0, 30, 60, 90, and 120 days) on the a* and b* axes of the CIELAB color space.

The flesh colors of the (I) and (A) cultivars during storage remained in yellow/pale tones, without major variations throughout the experiment. The actual flesh colors in RGB can be observed in Figure 6.

## 4 Discussion

Although linear models are often used to describe temporal trends, the occurrence of higher‐order fits for certain variables can be attributed to the dynamic and multifactorial nature of biological systems (Zhang et al. 2017). In postharvest studies, fluctuations in the evaluated parameters are expected due to physiological, metabolic, and environmental factors that continuously affect the product during storage (Pott et al. 2020; Lara and Fontanilles 2024), resulting in nonlinear behavior over time.

The behavior of SS content throughout storage confirms the influence of metabolic transformations on bulb composition, as described by Chitarra and Chitarra (2005), who associate the initial accumulation of these compounds with starch hydrolysis and the conversion of complex sugars into simple ones, followed by a gradual reduction due to respiration and tissue senescence. Similar results have been reported by Resende et al. (2013) and Beckles (2012), who highlight the degradation of sugars during the progression of physiological maturity and storage. When compared to literature data, the average values observed in this study align with those reported by Bessa et al. (2017), who recorded levels above 30 °Brix for the cultivars “Roxo Pérola de Caçador” and “Hozan” stored under ambient conditions, and by Almeida et al. (2022), who observed a variation between 35.70 and 39.67 °Brix for different cultivars stored in a controlled environment. These variations are attributed mainly to genetic differences between cultivars, the degree of maturity at harvest, and storage conditions, especially temperature and relative humidity (Souza and Macedo 2009). Thus, the fluctuations recorded in this work reflect the expected physiological behavior for bulbs stored for prolonged periods, demonstrating that the maintenance of SS content depends heavily on the interaction between genotype and environmental conditions.

Regarding sample acidification, cultivar (I) exhibited behavior closer to the pattern described by Bessa et al. (2017), characterized by a gradual increase in TTA and a continuous decrease in pH throughout storage. This behavior highlights the inverse relationship between acidity and pH, as reported by Chitarra and Chitarra (2005), who emphasize the role of acidity in maintaining the flavor and chemical stability of horticultural products. According to Lucena et al. (2016), higher acidity values confer better quality for garlic intended for processing, reinforcing the potential of cultivar (I) under the evaluated conditions. On the other hand, cultivar (A) exhibited a behavior distinct from the expected pattern, with a reduction in acidity and pH values until approximately 30 days of storage, followed by an increase in later periods, indicating a nonlinear trend. This variation may be associated with the buffering capacity of garlic paste, which allows fluctuations in TTA without significant changes in pH (Chitarra and Chitarra 2005). Furthermore, environmental factors may have contributed to this behavior. At 30 days of storage, a possible increase in ambient relative humidity may have intensified the bulb respiration process. Consequently, as organic acids constitute respiratory substrates, this process led to the degradation of these acids, resulting in reduced acidity (Berno et al. 2014).

Garlic Pg, measured by pyruvic acid concentration, was significantly influenced by cultivar and storage time. The behavior observed for cultivar AB aligns with the observations of Lima et al. (2019) and Nassur et al. (2020), who reported a gradual increase in Pg with advancing storage, related to the accumulation of sulfur compounds. Conversely, cultivar (I) showed lower initial values, with a slight increase up to 60 days and a subsequent sharp decrease at 90 and 120 days. This reduction behavior may be associated with the volatilization of pyruvic acid and the advancement of the WI, as highlighted by Bessa et al. (2017), who identified a trend of decreasing Pg during storage, although without statistical significance in their experiment. Comparing the obtained pyruvic acid levels, the average values recorded for the (A) cultivar, which ranged from 29.20 to 42.72 µmol mL^−1^ over the 120 days of storage, were close to those reported by Almeida et al. (2022) for cultivars such as “Centralina A” (30.02 µmol mL^−1^), “Branco Mineiro PI” (42.52 µmol mL^−1^), and “Branco Mineiro CB” (36.20 µmol mL^−1^). In contrast, the (I) cultivar exhibited lower levels, between 4.95 and 14.05 µmol Ml^−1^, characterizing lower Pg intensity and a greater reduction of this attribute during storage.

FML showed an increasing trend throughout storage, a result consistent with the advancement of bulb transpiration and respiration processes, as described by Chitarra and Chitarra (2005). The linear increase observed in both cultivars indicates that dehydration is inevitable even under controlled conditions, reflecting the gradual reduction in bulb moisture and fresh matter. Similar results were reported by Carvalho et al. (1991) and Bessa et al. (2017), who also observed a progressive increase in mass loss with prolonged storage. Cultivar AB exhibited slightly higher values than cultivar (I), suggesting greater susceptibility to water loss, possibly related to structural or physiological differences between the cultivars. Furthermore, it is possible that the (I) cultivar, after the curing process, had a higher moisture content in the bulb tunics compared to the (A) bulbs, resulting in greater mass losses during the storage period, since storage involves a deterioration process intensified by higher moisture content, which accelerates respiration.

The increasing mass loss observed during storage demonstrates the gradual reduction of water and fresh matter in the bulbs, reflecting the physiological processes of transpiration and respiration (Nassur et al. 2020; Oliveira et al. 2004). This dynamic is closely related to the variation in SS, since the mobilization and conversion of complex carbohydrates into simple sugars depend on the water and physiological status of the bulbs (Carvalho et al. 1991). Nevertheless, the final values observed, close to 6% at 120 days, indicate that the storage conditions adopted were adequate for maintaining postharvest quality, as the losses remained within acceptable limits for products stored for long periods.

The analyses indicated that both storage time and genotype influenced the WI, with a progressive trend of clove deterioration over the evaluated period. Despite the high data variability, the linear regression and LOESS adjustment show that cultivar (AB) exhibits greater physiological stability, while (I) demonstrates marked susceptibility to loss of firmness and the formation of shriveled cloves. These results reinforce that prolonged storage, especially in uncontrolled environmental conditions, compromises the commercial quality of garlic. Previous research underscores that time and storage conditions are critical for postharvest quality: Botrel et al. (2015) recorded 23.13% wilting in (I) after 410 days in an unpackaged environment, a value consistent with that observed here at 120 days, suggesting that lack of environmental control accelerates the defect. Meanwhile, Soares et al. (2016) reported 4.23% WI for (A), compatible with the levels in (A) up to 60 days in the present study, indicating greater resilience in this cultivar. Beyond storage variables, agronomic factors at harvest must also be considered. According to Luengo et al. (1996), the WI is related to the percentage of dry leaves on the plant at harvest, with the ideal range being between 59.4% and 71.6% dry leaves. In the present study, however, harvesting occurred during a period of intense rain, which prevented adequate field dehydration of the plants, compromising the optimal point of physiological maturity of the bulbs. This condition may have negatively affected both mass accumulation and susceptibility to wilting during storage, especially observed at the peak at 90 days, when there was a combination of low relative humidity (∼50%) and high temperatures (> 27°C), factors that aggravate postharvest stress.

In the multivariate analysis (PCA), differences observed between cultivars indicate that the postharvest dynamics of garlic are genotype‐dependent (Mota et al. 2004), reflecting variation in physiological responses during storage (El‐Mesery et al. 2025). This behavior may be associated with differences in the chemical composition and metabolic regulation of the bulbs, as reported in the literature.

For both cultivars, the observed associations indicate that WI is directly related to dehydration and, consequently, to tissue alterations that induce stress, being considered one of the main factors responsible for the reduction in postharvest quality of garlic under these conditions. These changes include shifts in chemical composition, degradation of bioactive compounds, and variations in attributes such as Pg and pH, reflecting the complex physiological response of the bulb to storage (Bessa et al. 2017; Akan et al. 2022; Prakash and Prasad 2023).

In contrast to the findings of Berno et al. (2014), who reported a decline in Pg during storage associated with increased WI and bulb deterioration, the results of the present study indicate that, for the Amarante cultivar, Pg was more closely related to FML than to structural changes. This pattern suggests that Pg may be more strongly linked to stress‐induced metabolic responses—particularly those associated with water deficit—than to the progression of deterioration itself (Carvalho et al. 1991). Thus, variation in this attribute appears to depend not only on storage duration but also on the intensity and type of stress to which the bulbs are subjected.

For cultivar (I), the WI proved to be an important indicator of deterioration, being associated with loss of turgidity and tissue disintegration alongside FML, which are typical features of advancing senescence. Thus, an increase in WI may indicate impairment of commercial quality, even in the absence of marked changes in certain physicochemical parameters, highlighting the importance of jointly evaluating physical and chemical attributes in postharvest quality assessment. Although WI represents only one of the evaluated parameters, it is highly relevant because it is classified as a quality defect under the current regulations of the Brazilian Ministry of Agriculture. This defect is characterized by moisture loss and depletion of internal clove content, resulting in shriveled, dry cloves with no commercial value (Brasil 2022; Luengo et al. 1996).

PCA also revealed a positive association between pH and TTA in cultivar (A). Although from a chemical standpoint, an inverse relationship between pH and acidity is commonly expected (Bessa et al. 2017), this pattern was not observed in the present study, as also noted for cultivar (I). This behavior may be attributed to the complex nature of some foods, such as garlic, in which TTA reflects the total amount of acids (both ionized and non‐ionized), whereas pH represents only the concentration of free H^+^ ions. Therefore, variations in the composition of organic acids—particularly in terms of their strength and degree of dissociation—as well as the buffering capacity of the matrix, may lead to increases in total acidity without a proportional decrease in pH. In this context, PC2 for cultivar (A) appears to reflect not only the quantity of acids present but also differences in acid composition and their behavior in solution, explaining the observed positive correlation (Sadler and Murphy 2010).

The distinct patterns of variation between cultivars indicate differentiated physiological responses, with direct implications for storage performance and shelf life. Overall, PCA proved to be a valuable tool for integrating and interpreting the data complexity, enabling a better understanding of how multiple quality attributes interact and contribute to the observed responses across cultivars and storage periods.

The stability of chroma and hue angle observed in the (A) skins suggests maintenance of the characteristic coloration throughout storage, which may indicate good preservation of the pigments responsible for the tone. In the case of the (I) cultivar, the greater color saturation and the hue closer to red may be associated with the sun‐curing process used, a practice that, according to Almeida et al. (2025), significantly influences bulb color parameters. This type of treatment tends to intensify pigmentation and promote shifts in tone, resulting in more reddish colorations than the originally expected purple. Other factors, such as pH variations, pigment oxidation, and interactions with other compounds, may also contribute to these deviations. According to Silva et al. (2019), in a study with grape anthocyanin extracts as pH indicators, anthocyanins, responsible for the purple hue, can be gradually degraded or shifted to forms associated with lower apparent pH, promoting a reddish coloration during storage. Factors such as subtle pH changes in the tissue, pigment oxidation, and interaction with co‐pigments may also have influenced this tonal alteration (Albarici et al. 2006; Lopes et al. 2007; Sadler and Murphy 2010).

Thus, the flesh of the Amarante cultivar maintained a stable light‐yellow hue, whereas Ito showed a slight decrease in hue at 90 days, suggesting a subtle tendency toward darkening, with no evidence of greening throughout storage. Greening is associated with reduced garlic quality and occurs more intensely after tissue disruption, as in processed garlic, but may also occur in fresh, intact cloves. This process is linked to enzymatic reactions involving sulfur‐containing compounds, in which alliinase and the γ‐ glutamyl transpeptidase (GGT), convert peptides of γ‐glutamil into sufoxides of S‐alquenil‐L‐cysteine (such as isoalliin/1‐propenyl‐CSO), which are subsequently transformed into thiosulfinates that give rise to green pigments (De Iseppi et al. 2021; Li et al. 2008; Li, N. et al. 2018). Greening is favored by low temperatures (0°C–13°C), which stimulate both isoalliin formation and GGT activity, increasing the availability of reactive substrates. Conversely, higher temperatures inhibit these metabolic pathways (Zhang et al. 2013; Li et al. 2008; Lu et al. 2023). Therefore, since temperatures remained above 20°C throughout the experimental period, this condition likely limited the formation of compounds responsible for green coloration, contributing to the preservation of chromatic integrity in the samples.

## 5 Conclusion

The Amarante and Ito cultivars showed similar behavior throughout storage regarding mass loss and WI, with a progressive increase in these parameters.

No changes were observed in skin and flesh color in either cultivar.

The Amarante cultivar exhibited lower deterioration over the storage period and, therefore, shows greater storage potential than the Ito cultivar. However, according to the parameters established by Brazilian legislation, storage of the Ito cultivar is recommended for up to 60 days, while the Amarante cultivar can be stored for up to 90 days without compromising its physicochemical and colorimetric qualities.

## Author Contributions

**Juliana Araújo da Silva**: conceptualization, methodology, data curation, investigation, visualization, writing – original draft, writing – review and editing. **Maiara Costa Silva**: conceptualization, methodology, data curation. **Marília Alves Brito Pinto**: validation, writing – review and editing, software, formal analysis. **Raquel Cardoso Guimarães**: data curation, investigation. **Luís Vicente Lima Teixeira**: data curation, investigation. **Sabrina Rocha Silva**: data curation, investigation. **Quelmo Silva de Novaes**: project administration, supervision, resources. **Gisele Brito Rodrigues**: conceptualization, methodology, validation, visualization, writing – review and editing, formal analysis, project administration, resources, supervision.

## Conflicts of Interest

The authors declare no conflicts of interest.
